# Mechanical Behavior of Five Different Morse Taper Implants and Abutments with Different Conical Internal Connections and Angles: An In Vitro Experimental Study

**DOI:** 10.3390/jfb15070177

**Published:** 2024-06-28

**Authors:** Claudia Caballero, Fernando Rodriguez, Guillermo Castro Cortellari, Antonio Scarano, Juan Carlos Prados-Frutos, Piedad N. De Aza, Gustavo Vicentis Oliveira Fernandes, Sergio Alexandre Gehrke

**Affiliations:** 1 Department of Implantology, Bioface/Postgrados en Odontología/Universidad Catolica de Murcia, 11100 Montevideo, Uruguay; claudiacaballero269@gmail.com (C.C.); rodrigueznando@hotmail.com (F.R.); guile237@gmail.com (G.C.C.); 2 Department of Innovative Technologies in Medicine & Dentistry, University of Chieti-Pescara, 66013 Chieti, Italy; ascarano@unich.it; 3 Department of Medical Specialties and Public Health, IDIBO Group (High Performance Group in Research and Development of Biomaterials in Dentistry), Rey Juan Carlos University, 28300 Madrid, Spain; prof.prados@gmail.com; 4 Department of Bioengineering, Universidad Miguel Hernandez de Elche, 03202 Alicante, Spain; piedad@umh.es; 5 Missouri School of Dentistry & Oral Health, A. T. Still University, St. Louis, MO 63104, USA; 6 Department of Biotechnology, Universidad Católica de Murcia (UCAM), 30107 Murcia, Spain

**Keywords:** abutment screw detorque, cone angle, disconnection force, mechanical load, Morse taper connections

## Abstract

The present study evaluated the mechanical behavior of five designs of Morse taper (MT) connections with and without the application of loads. For this, the detorque of the fixing screw and the traction force required to disconnect the abutment from the implant were assessed. A total of 100 sets of implants/abutments (IAs) with MT-type connections were used, comprising five groups (*n* = 20/group): (1) Group Imp 11.5: IA sets with a cone angulation of 11.5°; (2) Group SIN 11.5: with a cone angulation of 11.5°; (3) Group SIN 16: with a cone angulation of 16°; (4) Group Neo 16: with a cone angulation of 16°; and (5) Group Str 15: with a cone angulation of 15°. All sets received the torque recommended by the manufacturer. After applying the torque, the counter torque of the fixing screws was measured in ten IA sets of each group without the application of cyclic loads (frequencies ≤ 2 Hz, 360,000 cycles, and force at 150 Ncm). The other ten sets of each group were subjected to cyclic loads, after which the detorque was measured. Afterwards, the force for disconnection between the implant and the abutment was measured by traction on all the samples. The untwisting of the abutment fixation screws showed a decrease in relation to the initial torque applied in all groups. In the unloaded samples, it was found to be −25.7% in Group 1, −30.4% in Group 2, −36.8% in Group 3, −29.6% in Group 4, and −25.7% in Group 5. After the applied loads, it was found to be −44% in Group 1, −43.5% in Group 2, −48.5% in Group 3, −47.2% in Group 4, and −49.8% in Group 5. The values for the IA sets were zero for SIN 16 (Group 3) and Neo16 (Group 4), both without and with loads. In the other three groups, without loads, the disconnection value was 56.3 ± 2.21 N (Group 1), 30.7 ± 2.00 N (Group 2), and 26.0 ± 2.52 N (Group 5). After applying loads, the values were 63.5 ± 3.06 N for Group 1, 34.2 ± 2.45 N in Group 2, and 23.1 ± 1.29 N in Group 5. It was concluded that in terms of the mechanical behavior of the five designs of MT IA sets, with and without the application of loads, the Imp 11.5, SIN 11.5, and Srt 15 groups showed better results compared to the SIN 16 and Neo 16 groups, showing that lower values of cone angulation increase the friction between the parts (IA), thus avoiding the need to maintain the torque of the fixing screw to maintain the union of the sets.

## 1. Introduction

The technique of replacing missing teeth with implant-supported dentures has become highly used and predictable due to many years of research and development, boasting high long-term survival rates [1,2]. However, several studies have shown that late failures in implant rehabilitation can occur predominantly during the first few years [3,4,5,6]. Complications with the dental implant can happen due to infections of the peri-implant tissues and occlusal overload, affecting the local peri-implant bone tissue and, in the worst-case scenario, leading to consequent implant mobility and loss [4,7]. On the other hand, complications associated with the implant–abutment (IA) junction are also frequent, such as loosening and fracture of the abutment fixation screw, cracks, and/or superficial fractures between the metallic structure and the layers of coating material (acrylic or ceramic) [8,9]. The IA junction is one of the sites most susceptible to failure. This area receives the functional forces of occlusion, which are distributed across the dental implant platform and bone. Hence, any tension or deformation of the prosthesis caused by misfit or local instability could lead to technical complications [10].

In screwed fixed crowns, a frequent problem is the control of the appropriate torque according to the screw and connection types in order to apply adequate preload. It is the torque applied to the fixation screw that develops a compressive tightening force between the IA sets, maintaining the stability of the components [11,12]. The correct torque method is required to determine the preload to be imposed on the sets, but some preload loss (dispersion) is expected [13,14]. The torque value applied to the screw has a unique relationship with the preload generated by the screw on the apical third of the implant, which is directly influenced by the design of the abutment/implant interface, the type of retention screw, and the torque value applied [13]. These factors interfere with the biomechanical stability of the IA interface, which is still dependent on the tolerance between components, the freedom for rotation, and the adjustment precision [15]. However, preload values are determined by the manufacturer, considering the strength limits of the screw of each system.

Due to mechanical problems, new designs for the prosthetic interface have been developed, highlighting the internal connection to the implant, such as the internal hexagon (HI) and the Morse taper (MT). Originally created by the mechanical tool industry, the term Morse taper describes a fitting mechanism where two elements seek close contact through friction. This mechanism occurs when the conical pillar is installed in a conical cavity, generating friction that becomes significant due to the parallelism of these two structures. In this type of connection, where the Morse angle is determined according to the mechanical properties of each material, there is a relationship between the angle values and the friction of the parts [16].

New designs for MT implant systems were developed with the aim of improving the IA union, meeting the needs of prosthetic rehabilitation, and improving the mechanical behavior during the application of functional masticatory loads, given the hypothesis that there is no difference in the mechanical behavior of one- or two-piece abutments [17]. In the MT connection, the abutment is joined to the implant by contact (friction), with mechanical locking between the implant cone and the prosthetic component cone. This type of locking allows the abutment to achieve good stability even when losing part of the torque (preload) applied, reducing the possibility of micromovement during functional loads, not overloading the screw, and reducing the incidence of loosening and/or fracture [8,9,18]. The IA connection influences how the system fails, with each system’s characteristics being a relevant factor for clinical indication [18,19]. Furthermore, the stress concentration tends to decrease when the internal surface area of the system increases [20].

During the application of the cyclic load on the abutment installed on the MT implant, intrusion and/or settlement occurs at the IA interface, increasing the interlocking between the IA set [15,21,22]. Thus, more close contact between the surfaces of the two pieces will occur through juxtaposition until there is no more displacement. Therefore, this better fit between the pieces favors joint action, improving the distribution of chewing forces that affect the IA set [23]. The micromovements between the implant and the prosthetic component could lead to the formation of microgaps at that IA junction. Therefore, the greater overlap between the parts and the consequent increase in the Morse effect are positives for the IA interface’s sealing and the IA assemblies’ stability [24].

Currently, there are different designs of MT fittings on the market between implant brands. They vary in terms of the extent of contact between the abutment and the implant and the angle of the walls. Thus, the present study sought to evaluate the mechanical behavior of five different MT connection designs with and without the application of loads. For this, the detorque of the fixing screw and the traction force required to disconnect the abutment from the implant were evaluated. The positive hypothesis was that the increased angulation in the design of the cone that joins the pieces (IA) would reduce the friction, increasing the need for the fixing screw to keep the pieces together; the null hypothesis was that no difference would be observed for different angulations for the parameters tested.

## 2. Materials and Methods

**Figure 1:**
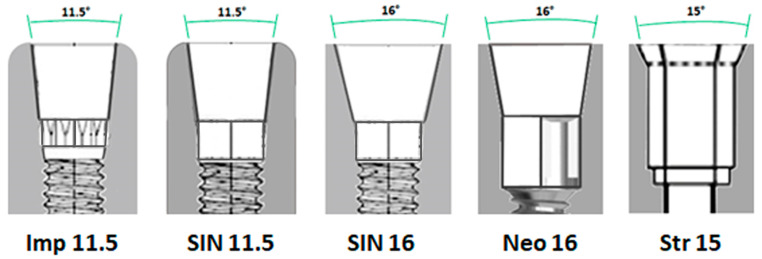
Schematic images of the internal connection and angulation of each IA set used.

One hundred IA sets of internal Morse taper connections (*n* = 100) with five different types of design were tested. They formed 5 experimental groups (*n* = 20 sets per group): In Group 1—Imp. 11.5 (G1), DuoCone implants and BaseT abutments were used (Implacil De Bortoli, São Paulo, Brasil). The implants used were 4 mm in diameter and 9 mm in length. The internal connection had a double cone separated by a 12-position index between the two cones, with an angulation of 11.5°. In Group 2—SIN 11.5 (G2), Strong SWC implants and Pilar Universal abutments were used (Sistema Nacional de Implantes, São Paulo, Brazil). The implants were 3.8 mm in diameter and 10 mm in length. The internal connection had a cone and a hexagonal index in its final portion, with an angle of 11.5°. In Group 3—SIN 16 (G3), Strong SW implants and Pilar Universal abutments were used (Sistema Nacional de Implantes, São Paulo, Brazil). The implants used were 3.8 mm in diameter and 10 mm in length. The internal connection had a cone and a hexagonal index in its final portion, with an angle of 16°. In Group 4, Neo 16 (G4), Helix GM implants and titanium base abutments (Neodent/Straumann, Curitiba, Brazil) were used. The implants used were 4 mm in diameter and 10 mm in length. The internal connection had a cone and a hexagonal index in its final portion, with an angle of 16°. In Group 5—Str 15 (G5), Helix GM implants and Regular CrossFit^®^ abutments (Straumann, Basel, Switzerland) were used. The implants were 4.1 mm in diameter and 10 mm in length. The internal connection featured a mechanical locking friction adjustment connection with four grooves and 15° angulation. Figure 1 shows a schematic image of the internal connection of the IA sets.

**Figure 2:**
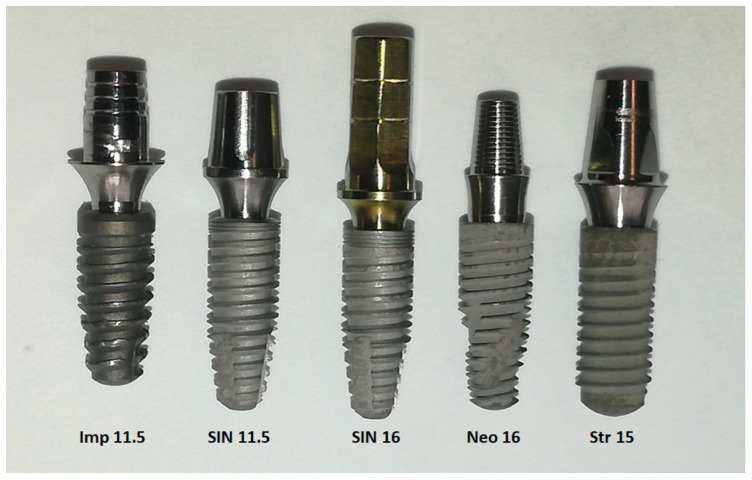
IA sets for the 5 groups evaluated in the present study, sorted from the left to the right according to group.

All abutments used were two-piece ones (abutment and fixation screw). Figure 2 shows images of the IA sets used in the present study.

Initially, all IA assemblies were joined and torqued to the values recommended by each manufacturer: 20 Ncm (G1), 10 Ncm (G2), 20 Ncm (G3), 20 Ncm (G4), and 35 Ncm (G5). After 10 min, all sets were retorqued [21,22]. After one hour, 10 IA sets from each group were taken to computerized torquemeter equipment (CME-30Nm, Técnica Industrial Oswaldo Filizola Ltd.a., São Paulo, Brazil) to measure the torque values of the fixing screw. Then, the fixing screws were completely unscrewed, and each set was taken to a universal machine (model AME-5kN; Técnica Industrial Oswaldo Filizola Ltd.a., São Paulo, Brazil) with a 0.5 mm/min crosshead speed for tensile testing, measuring the force required to separate the parts (implant and abutment).

**Figure 3:**
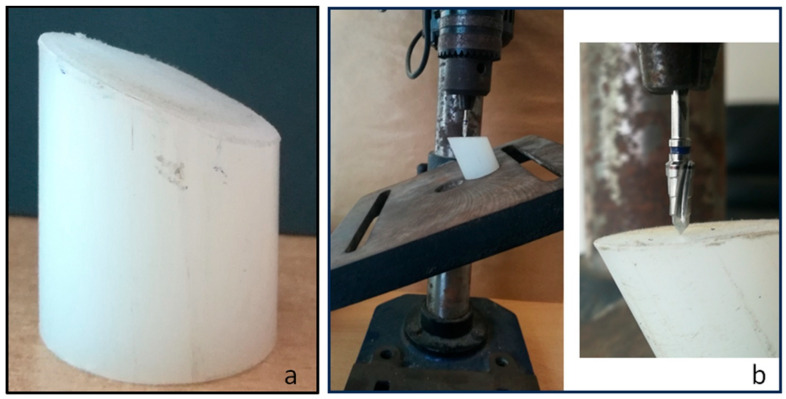
(**a**) A cylindrical block of polyurethane was cut to 30 mm in diameter and 30 mm in height, with one side being cut at an angle of 30 ± 2°. (**b**) The cylindrical block was positioned on an angled table at 30° to perform drilling using the drill sequence indicated for each implant brand, obtaining the ideal load direction.

Fifty cylindrical polyurethane blocks were cut to 30 mm in diameter and 30 mm in height, with one side being cut at an angle of 30 ± 2° (Figure 3a). Ten implant samples from each group were inserted into the blocks, leaving 3 mm outside the base for all the implants. The implant position (angulation and level) was determined according to recommendations from the International Organization for Standardization (ISO) 14.801:2016 [25]. To insert the implants, each cylindrical block was positioned on an angled table of 30° to perform drilling using the drill sequence indicated for each implant brand, thus obtaining the ideal load direction (Figure 3b).

**Figure 4:**
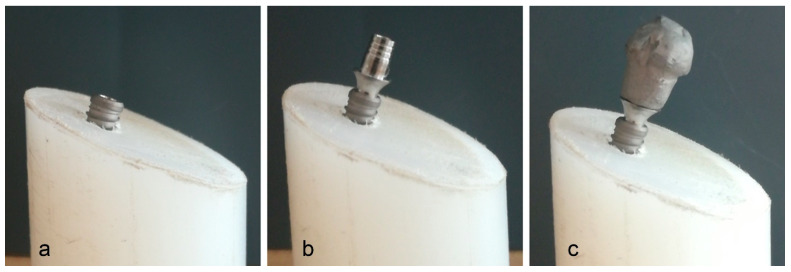
(**a**) Implants were placed into the blocks. (**b**) Abutments were positioned on the implants, receiving the corresponding torque and retorque. (**c**) A metallic hemisphere was cemented on the abutments.

The implants were then inserted into the blocks. The abutments were positioned on them, receiving the corresponding torque and retorque. A metallic hemisphere was similarly fabricated for all sets and cemented on the abutments. To manufacture the hemispheres, a CAD/CAM (computer-aided design/computer-aided manufacturing) system was used, with one abutment from each group being scanned and the shape of the hemisphere adapted onto each abutment; then, the hemispheres were printed in wax. Finally, all hemispheres were included in a coating and cast in cobalt chromium. To cement the hemispheres, a temporary cement (Temp-Bond, Kerr, USA) with low adhesion was used, enabling the de-cementation of the hemispheres without affecting the stability of the abutments. Then, a force was applied from a plane surface to avoid reducing the magnitude of the applied load (Figure 4a–c).

The specimens were kept submerged in water at 37 ± 2 °C during the test. The test was carried out at frequencies of ≤ 2 Hz. Then, 360,000 cycles of mechanical loading with controlled nonaxial force at 150 Ncm were applied using a mechanical cycler (Biocycle V2, BioPDI, São Carlos, Brazil). After applying the cyclic loads, the samples were taken to the computerized torquemeter equipment (CME-30Nm, Tecnica Industrial Oswaldo Filizola Ltd.a., São Paulo, Brazil) to measure the detorque values of the fixing screw. Therefore, the fixing screws were completely unscrewed, and each set was taken to a universal machine (model AME-5kN; Técnica Industrial Oswaldo Filizola Ltd.a) with a 0.5 mm/min crosshead speed for tensile testing, measuring the tensile strength value (TSv) necessary to separate the parts (implant and abutment).

G*Power software (Heinrich-Heine-Universität Düsseldorf, Düsseldorf, Germany) was used to calculate the effect sizes, considering the F tests (fixed effects, omnibus, one-way), of the five groups with means and standard deviations, with 10 samples each. In the first situation (without loading) at a 5% level of significance and 95% power, considering the maximum level of standard deviation as 1.44 Ncm, the effect size generated was 4.26. In the second situation (with loading) at a 5% level of significance and 95% power, considering the maximum level of standard deviation as 1.5 Ncm, the effect size was 2.58.

### Statistical Analysis

To analyze the torque of the fixing screws with and without load application, the values obtained were used to calculate the percentage of loss in relation to the initial torque applied, which were the values recommended by the manufacturers. GraphPad Prism 5.01 software (GraphPad Software Inc., San Diego, CA, USA) was used for the statistical analysis of the torque values for the fixing screws and the traction needed to disconnect the IA assemblies; they were considered statistically significant if *p* < 0.05. After the Kolmogorov–Smirnov test for normality, Bonferroni’s multiple comparison tests detected possible differences between groups.

## 3. Results

The assessment of behavior by comparing different designs of internal conical connections provides an indication of their behavioral characteristics during their placement for a masticatory function. It can contribute to a successful outcome after the implant’s clinical placement. The results showed important differences between the groups evaluated. The detorque of the screws for the abutment fixation showed considerable loss in all groups compared to the initial torque applied. Table 1 presents the applied torque values, following the recommendation of the manufacturers, the measured detorque values, and the percentage value calculated in each group in both the tested conditions (without and with load application). Without the application of loads, the data collected showed significant differences between the groups Impl 11.5 (G1) versus SIN 16 (G3) (*p* = 0.0045) and SIN 16 (G3) versus Str 15 (G5) (*p* = 0.0039); the other groups did not show statistically significant differences. After applying cyclic loads, the Kolmogorov–Smirnov test showed a normal distribution, and no differences were detected among the groups (ANOVA *p* = 0.2697).

In the traction test, for the disconnection of the IA sets, higher values were found in the abutment groups with angulations of 11.5° (G1 and G2) and 15° (G5) compared to the groups with angulations of 16° (G3 and G4) before and after the application of cyclic loads. Table 2 presents the values measured in the five groups without and with the application of loads. Statistical comparisons were carried out among the three groups that presented values greater than zero for tensile strength (Imp 11.5 [G1], SIN 11.5 [G2], and Str 15 [G5]). Table 3 presents the statistical analysis achieved.

## 4. Discussion

Abutments have been used to connect dental implants with the prosthetic superstructure, permitting patient rehabilitation. Typically, they are screwed to the dental implant, like the abutments used in this study. They can be customized or prefabricated and made of titanium, zirconium oxide, gold, or aluminum oxide (the material of the abutments herein used) [26,27]. There are two types of IA connections: internal and external. This depends on whether the geometric feature extends above or below the platform’s coronal surface. In internal taper-connected implant systems, the tightening torque depends not only on the screw height but also on the wedging effect caused by the subsidence of the tapered abutment, while the internal inclination of the device mainly bears the load. Therefore, the load on the abutment screw is lower compared to the external connection. The MT system promotes torque amplification through friction between the internal implant surface and the tapered abutment, resulting in high stability [28,29]. Moreover, some authors [30] compared the prevalence of screw loosening between external IA connections and internal connections, with respective results of 4.8% and 1.2% over a 5-year follow-up. Thus, the goal of this study was to assess the mechanical behavior (the detorque of the fixing screw and the traction force required to disconnect the abutment from the implant) of five different MT (internal) connection designs with and without the application of loads.

Comparing different designs of internal conical connections can help predict the masticatory function behavior due to the loss of screw torque, which might be caused by the axial and nonaxial forces that the implant superstructure is subject to. According to the mechanical principles of levers, nonaxial forces might be provoked by the implant’s prosthetic part, which must be considered and adequately observed by clinicians; in this study, a similar superstructure was used in all the groups to avoid variations. In this study, we measured the initial torque, detorque, and tensile strength values of five different MT implants with and without loads applied. Our results showed important differences among the groups evaluated. The distortion of the screws for the abutment fixation in all groups led to considerable torque loss compared to the initial torque. Therefore, without the application of loads, there were significant differences between Impl 11.5 (G1) versus SIN 16 (G3) and SIN 16 (G3) versus Str 15 (G5). According to the mechanical principles of screws, the application of torque causes stretching and tension, which create preload forces on the screw [31]. This force also refers to the longitudinal axial force generated between the threads of the abutment screw and the internal components of the implant [32]. It should be maintained and minimized to prevent loosening of the connection [33].

It is important to remember that the higher the number of cycles used, the greater the initial torque loss value [34]. Thereby, this analysis is highly important owing to screw loosening or screw fractures being the most common technical complications of implant-supported prosthetic rehabilitation [30,35].

The pre-tightening force was positively related to the screw-tightening torque value [13]. Furthermore, in this study, we kept the torque values recommended by the manufacturers, specifically 20 Ncm (G1), 10 Ncm (G2), 20 Ncm (G3), 20 Ncm (G4), and 35 Ncm (G5). The ideal pre-stress reported in the literature is approximately 60–80% of the material yield strength [36]. Only 10% of the torque is converted into preload force, and the remaining 90% is used to overcome the friction between mating surfaces [31,32]. Even in the absence of external force, the preload loss is observed within the first 2–3 min [29,33] or 15 h after tightening [13].

Screw loosening happens when the external separation force exerted on the IA connection is greater than the clamping force holding the implant and abutment tightly together [36]. Any forces applied to the implant superstructure are followed by tensile and compressive stresses at the IA connections [36], resulting in micromovements between the IA connection, which might terminate with screw torque loss complications [23,24]. The incidence of abutment screw loosening for single crowns is 12.7%, and it is 6.7% for splinted crowns [37]. The most common clinical complications of abutment screw loosening include gingivitis and screw fractures [38]. To reduce these problems, several solutions have been proposed, including retightening abutment screws after initial tightening and applying increased torque [39]. Despite numerous studies, the exact cause of abutment screw loosening is unknown [40,41]. It may be attributed to insufficient tightening torque, incorrect implant positioning, an inappropriate occlusal surface or crown anatomy, poor abutment and crown fit, microleakage at the IA interface, inappropriate screw design/materials, and occlusal forces. Any oversizing can be considered a cause of abutment screw loosening [38].

Bagegni et al.’s [42] results contrasted with an in vitro study [43] that found that the mechanical strength of the screwless MT (3°) IA connection is lower than that of the screw-retained ones (all samples of the screwless Morse taper implants failed to survive the planned dynamic loading (1.2 × 10^6^ loading cycles); they all fractured after less than 100,000 loading cycles when a load of 120 N was applied at 30° to the long axis of the implants). In Bagegni et al.’s study [42], all the screwless Morse taper implants survived 1.0 × 10^6^ cycles with a load of 100 Ncm. The only explanation to justify this high level of variation is that the implants in Ugurel et al.’s study [43] had a thinner implant headwall. Therefore, the screw-retained internal groups in that same study [43] exhibited early abutment and/or screw fractures. Those results were also different compared with the screw-retained implant group in Bagegni et al.’s study [42]. In our study, we included only screw-retained MT implants; we used a regular standard for frequencies of ≤2 Hz and 360,000 cycles of mechanical loading with a controlled nonaxial force of 150 Ncm. We did not find fractures, and the detorque with loadings was similar among all the groups, although the G3 (SIN 16) group without loads had a higher and more significant detorque than G1 and G5. These findings agree with Bagegni et al.’s results.

In Ebadian et al.’s study [44], the abutment screw detorque that used the minimum mean abutment screw torque value was found for the group with three 30 Ncm abutment screw torques, which underwent five-minute intervals of mechanical cycling; therefore, this methodology was different to that applied in this study. Moreover, the torque used here was recommended by the manufacturers and was lower than that applied in Ebadian et al.’s study [44] (30 Ncm), except for G5 (35 Ncm). So, as reported in the literature [44], less screw loosening is expected to occur when 35 Ncm of torque is applied to the abutment screw in this implant system. Nonetheless, after applying cyclic loads, no differences were detected among all the groups (*p* = 0.2697), neither with nor without load.

The tensile strength was statistically compared among the three groups that obtained values greater than zero (G1, G2, and G5). The values achieved for G3 and G4, both groups with 16° angulation, demonstrated that for this type of analysis, no tensile resistance was observed (the implant and abutment were easily separated). The angles for these groups were, respectively, 11.5°, 11.5°, and 15°. The worst result for this parameter among them was found in Group 1 (G1), with significantly lower results compared to G2 and G5 without and with loads (*p* < 0.0001 and *p* < 0.0002), and G5 had the best results without and with loads (*p* = 0.0009 and *p* = 0.0002).

The results obtained in this study should be carefully analyzed; their interpretation and clinical application should be approached with caution, given the differences in biological behavior between in vitro and in vivo scenarios. Moreover, the groups here studied had different Morse taper connection properties, besides the angulation, such as different abutment heights. The higher the IA contact surfaces, the higher might be the influence on the results. On the other hand, one of the implants with a greater IA contact surface (Neo 16 [G4] group) had no favorable outcomes, whereas the other group with more IA contact (Str 15 [G5]) did achieve good outcomes. New studies must be carried out to better evaluate this parameter.

## 5. Conclusions

It was possible to confirm the positive hypothesis proposed in this study and to conclude that the design of the internal connection influences the detorque of the fixing screw and the tensile strength of the IA sets. The implants with smaller angulations, 11.5° and 15° (groups Imp 11.5, SIN 11.5, and Str 15), showed better results compared to the implants with greater angulations, 16° (groups SIN 16 and Neo 16). These results demonstrate that lower values of cone angulation increase the friction between the parts (IA), hence avoiding the need to maintain the torque of the fixing screw to maintain the union of the sets.

## Figures and Tables

**Table 1:** Initial recommended torque (RT), measured detorque (DT), and the percentage of torque loss calculated for each group with and without load.

		Without Load		With Load	
Groups	RT	DT	Perc. diff.	DT	Perc. diff.
G1	20 Ncm	14.9 ± 1.44 Ncm	−25.7%	11.2 ± 1.44 Ncm	−44.0%
G2	10 Ncm	7.0 ± 0.87 Ncm	−30.4%	5.7 ± 0.81 Ncm	−43.5%
G3	20 Ncm	12.7 ± 1.28 Ncm	−36.8%	10.3 ± 1.15 Ncm	−48.5%
G4	20 Ncm	14.1 ± 1.32 Ncm	−29.6%	10.6 ± 1.08 Ncm	−47.2%
G5	35 Ncm	25.9 ± 1.42 Ncm	−25.7%	17.6 ± 1.49 Ncm	−49.8%

RT = recommended torque by the manufacturers; DT = detorque; Perc. diff. = percentage difference; G1—Imp. 11.5; G2—SIN 11.5; G3—SIN 16; G4—Neo 16; G5—Str 15.

**Table 2:** Mean and standard deviation of the tensile strength values for each group with and without load. Values are in Newtons (N).

Groups	Without Load TSv	With Load TSv
G1	56.3 ± 2.21 N	63.5 ± 3.06 N
G2	30.7 ± 2.00 N	34.2 ± 2.45 N
G3	0 N	0 N
G4	0 N	0 N
G5	26.0 ± 2.52 N	23.1 ± 1.29 N

TSv = tensile strength value; G1—Imp. 11.5; G2—SIN 11.5; G3—SIN 16; G4—Neo 16; G5—Str 15.

**Table 3:** Statistical comparison for the tensile strength values of the groups that presented values greater than zero with and without loads.

	Without Load			With Load		
Comparisons	Mean Diff.	*p*-Value	95% CI of diff	Mean Diff.	*p*-Value	95% CI of diff
G1 vs. G2	25.56	<0.0001	22.98 to 28.14	29.32	0.0002	26.60 to 32.04
G1 vs. G5	30.28	<0.0001	27.70 to 32.86	40.42	0.0002	37.70 to 43.14
G2 vs. G5	4.720	0.0009	2.144 to 7.296	11.10	0.0002	8.382 to 13.82

Mean Diff. = mean of difference; CI of diff = confidence intervals of difference; vs. = versus. G1—Imp. 11.5; G2—SIN 11.5; G3—SIN 16; G4—Neo 16; G5—Str 15.

## Data Availability

The original contributions presented in the study are included in the article, further inquiries can be directed to the corresponding authors.
